# A Novel Fragment Derived from Laminin-411 Facilitates Proliferation and Differentiation of Odontoblast-Like Cells

**DOI:** 10.1155/2018/9465383

**Published:** 2018-05-09

**Authors:** Jia Tang, Takashi Saito

**Affiliations:** ^1^Division of Biochemistry, Department of Oral Biology, School of Dentistry, Health Sciences University of Hokkaido, Hokkaido, Japan; ^2^Division of Clinical Cariology and Endodontology, Department of Oral Rehabilitation, School of Dentistry, Health Sciences University of Hokkaido, Hokkaido, Japan

## Abstract

The aim for the present study was to evaluate the* in vitro* effects of iMatrix-411 in odontoblast-like cells. To that end, iMatrix-411 was coated to both nontissue culture treated- (Non-PS) and tissue culture treated-polystyrene (TCPS) multiwells. MDPC-23 cells were seeded into noncoated (control) or coated wells. Optimal coating density and cell proliferation were assessed by cell counting kit-8 (CCK-8) at day two, day three, and day five. Osteo/odontogenic differentiation was evaluated by real-time RT-PCR and alkaline phosphatase (ALP) activity at days seven and eight, respectively. Calcific deposition of cells was visualized by alizarin red staining. Data were analyzed with post hoc Tukey HSD test (*p* < 0.05). Optimal coating density for iMatrix-411 was 8 *μ*g/cm^2^. Exposure of MDPC-23 cells to iMatrix-411 in either non-PS or TCPS significantly enhanced proliferative activity. iMatrix-411 elevated ALP activity in both types of culture plates. iMatrix-411 significantly increased the mRNA level of OCN, BSP, OPN, ALP, and DMP-1. Meanwhile, it enhanced the expression of several integrin subunits: ITGA1, ITGA5, ITGAV, ITGB1, and ITGB5. Finally, iMatrix-411 also accelerated the mineralization at day eight in Non-PS. The results indicated iMatrix-411 stimulates proliferation and favours differentiation of odontoblast-like cells.

## 1. Introduction

Calcium hydroxide (CH) is commonly used for capping exposed or nearly exposed pulp in an effort to initiate a healing process within wound site. However, its nine-year success rate (SR) is only 58.7% [1] and the SR tends to further decrease over time, primarily due to high alkalinity and poor adhesiveness of CH to dentine tissue. Moreover, when the pulp is exposed for protracted period, the possibility of pulp infection could not be ruled out. Under this circumstance, a more invasive treatment named root canal therapy (RCT) needs to be implemented. Although the treating technique and instruments have evolved dramatically over the years, endodontic failure and complications still occur following RCT and remain a major concern for dentists [2]. Furthermore, RCT sacrifices the sensory and forming ability for structural integrity of teeth. Therefore, it is of critical importance to maintain the integrity of dentine structure before infection reaches the pulp.

Laminin (LN) is a heterotrimer glycoprotein that contains *α*, *β*, and *γ* chains. Nomenclature for LN is based on its chain composition; for instance, LN-411 (or LN-8) comprises *α*4, *β*1, and *γ*1 chains. LN is a key component of basement membrane and is regulating a wide range of cellular activities such as proliferation [3, 4], migration [5, 6], and differentiation [7, 8]. Fragmentation of LN by proteolysis rendered seven types of distinct domains, namely, E3, T8, E8, C8-9, C1–4, P1, and E4 [9]. Among those, E8 is a fragment comprising the lower 35 nm of the long arm and lacking two distant COOH-terminal globular domains (G4 and G5, E3 fragment) [10]. It avidly binds with *α*6*β*1 integrin, a major LN receptor [11], as antibody against *α*6 chain intensively blocked the cell attachment to E8 but not to other matrix molecules.

We already demonstrated LN-111 (or LN-1) is an odontoblast-like cell adhesive and conducive to its differentiation toward hard tissue forming phenotype [12]. Unlike LN-111, LN-411 is expressed in vascular endothelial basement membrane [13]. It facilitates differentiation of umbilical cord mesenchymal stem cell differentiation into insulin-producing cells [14] and mediates the migration of T helper 17 cells (TH17) into central nervous system (CNS) by acting as a vascular ligand for CD146 (or melanoma cell adhesion molecule, MCAM) [15]. Moreover, the role of LN-411 in promoting tumor cell migration was confirmed in melanomas, gliomas, and carcinomas via *α*6*β*1 integrin, albeit less efficiently than LN-421 [16]. Recently, an E8 fragment (iMatrix-411) from LN-411 was purified. iMatrix-411 retains full binding activity toward integrins but lacks binding activity to other cell matrix components [13]. It induced the differentiation of human iPS into cholangiocytes by upregulating several cholangiocyte markers such as aquaporin 1, SRY-box9 (SOX9), Jagged 1 (JAG 1), and secretin receptor (SCTR) [17]. Despite the above findings, the function of LN-411 in odontoblasts received less attention and is far from clear.

Odontoblasts, located in periphery of pulp chamber, originate from dental papillae and are known to secret dentine matrix. In this experiment, we uncovered the precise roles of iMatrix-411 in the proliferation and differentiation of odontoblast-like cells. Moreover, we addressed the question whether cultivation of cells in different types of polystyrene impacts those parameters.

## 2. Materials and Methods

### 2.1. Cell Culture

MDPC-23 cells, generously provided by Professor Jacques E. Nör (University of Michigan), were maintained in Dulbecco's Modified Eagle Medium (DMEM, D5796, high glucose type, Sigma) containing 5% fetal bovine serum (FBS, 10270-106, Gibco) (maintenance medium: MM) at 37°C in a humidified atmosphere with 5% CO_2_. Media were refreshed every other day. For osteo/odontogenic induction, MM were changed into inducing media (IM) containing *β*-glycerophosphate (*β*-GP, 10 mM, 191-02042, Wako), ascorbic acid (AA, 50 *μ*g/mL, 013-19641, Wako), and dexamethasone (Dex, 100 nM, D2915, Sigma) on the day achieving confluence.

### 2.2. Coating of iMatrix-411

iMatrix-411 (Product No. 892041, Nippi) was coated into two types of polystyrene: nontissue culture polystyrene (Non-PS) and tissue culture polystyrene (TCPS). Gene expression of odonto/osteogenic markers and integrins and alkaline phosphatase activity of MDPC-23 cells were analyzed and compared on those non-PS or TCPS multiwell plates. Briefly, iMatrix-411 was diluted in phosphate buffer saline (PBS) to desired concentration and added to multiwell plates, incubated for 2 h under 37°C. After incubation, the coating solution was aspirated and cells were inoculated immediately without washing the wells. Coating volume for protein in 96-well plates (surface area: 0.32 cm^2^) and 12-well plates (surface area: 3.8 cm^2^) was 50 *μ*L/well and 400 *μ*L/well. Wells coated with PBS served as noncoated control (non-PS or TCPS).

### 2.3. Cell Counting Kit-8 (CCK-8) Assay

iMatrix-411 solution with various concentrations was coated into 96-well plates (Non-PS, 351172, Falcon & TCPS, 353072, Falcon) making the final density: 0.5, 1, 2, 4, 8, and 16 *μ*g/cm^2^. Cells were seeded into those surface-modified or nonmodified wells at the number of 1 × 10^3^/well. Cell viability was evaluated by addition of CCK-8 (Dojindo, Japan) into each well (10 *μ*L/well) and incubation for 1 h 45 min. Absorbance was read at the wavelength of 450 nm. For cell proliferation test, cells were seeded in the same manner with lower number (0.5 × 10^3^/well).

### 2.4. Alkaline Phosphatase (ALP) Activity

Cells were seeded at the number of 1.25 × 10^4^/well in 12-well plates (Non-PS, 351143, Falcon & TCPS, 353043, Falcon). IM were added on day five upon confluence. At day eight, cells were scraped from the culture plates and sonicated on ice for ten minutes. The cell lysates were centrifuged at 12,000 rpm, 4°C for 15 min, and supernatant was recovered to quantify the ALP activity (Wako) and protein amount (Pierce).

### 2.5. Quantitative Real-Time PCR (qRT-PCR) Analysis

MDPC-23 cells were seeded at the number of 1.25 × 10^4^/well and cultured with MM in 12-well plates to confluence on day five. At day five, MM including *β*-GP, AA, and Dex were added. Total RNA was extracted using Trizol reagent on day seven. After RNA extraction, qRT-PCR was performed as described previously [12]. The primer sequences are listed in Table 1. Gene expression levels were calculated as fold changes compared with noncoated control (Non-PS). *β*-Actin was taken to be internal control.

### 2.6. Alizarin Red Staining

MDPC-23 cells were inoculated at the number of 1.25 × 10^4^/well and cultured with MM in 12-well plates until day four. At day four, IM described above in cell culture were added to culture media. Calcific deposition of cells was visualized using alizarin red staining by day eight. Briefly, cell monolayer was rinsed once by PBS and fixed in 10% formalin neutral buffer solution (060-01667, Wako) for 20 min at room temperature. Thereafter, cells were washed once using distilled water and stained by alizarin red s solution (1%, pH 4.1, 011-01192, Wako) for about five minutes at room temperature. Staining solution was discarded and cell monolayer was washed by distilled water for 2 h. The stained mineralized nodules were photographed by an inverted digital camera (Canon) and quantified using cetylpyridinium chloride (CPC). The detailed quantification method was described elsewhere [18].

### 2.7. Statistical Analysis

Results were expressed as mean ± SD (standard deviation). Statistical analysis was conducted using post hoc Tukey's HSD test. A value of *p* < 0.05 was considered statistically significant.

## 3. Results

### 3.1. iMatrix-411 Elicits Optimal Cell Viability at the Coating Density of 8 *μ*g/cm^*2*^

**Figure 1:**
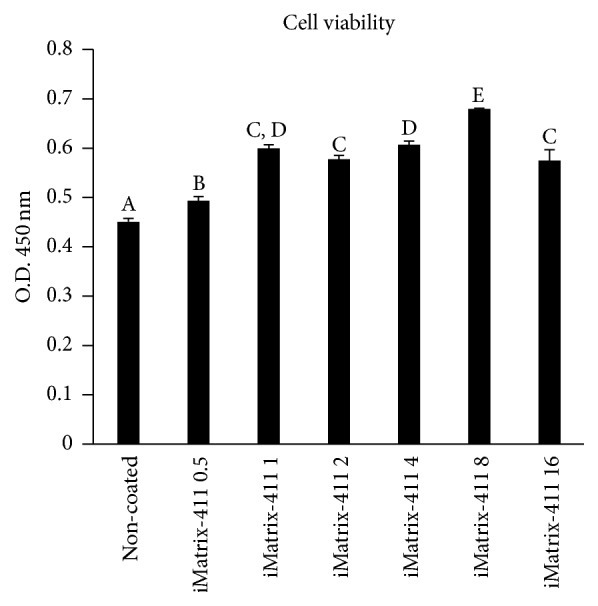
Cell viability test. The viability of MDPC-23 cells showed surface density-dependent increment trend when it reached 8 *μ*g/cm^2^. (A–E indicate significant differences between different characters, *p* < 0.05, post hoc Tukey HSD test).

A comparison of cell viability revealed that the optimal coating density for iMatrix-411 was 8 *μ*g/cm^2^ (Figure 1). The following experiments were conducted using this density.

### 3.2. iMatrix-411 Facilitates Early Adhesion and Spreading of MDPC-23 Cells

**Figure 2:**
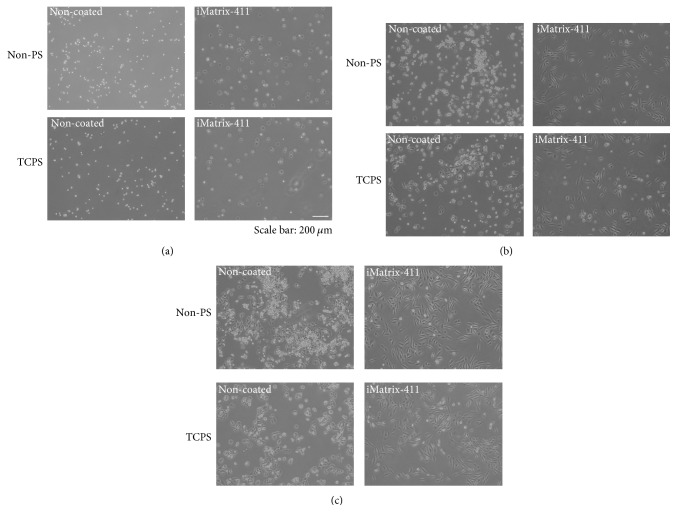
Morphology of MDPC-23 cells on iMatrix-411-coated Non-PS and TCPS at different time points: 1 h (a), 23 h (b), and 48 h (c). Scale bar: 200 *μ*m.

Visually, cells started to flatten as early as 1 h when they were inoculated to iMatrix-411-modified polystyrene surfaces (Non-PS and TCPS), while those on noncoated controls were still round, spot-like in shape (Figure 2(a)). There seems to be no difference of cell morphology between noncoated Non-PS and noncoated TCPS. At 23 h and 48 h, differences among surfaces were even more evident. Cells on iMatrix-411-modified non-PS or TCPS adopted spindle shape were elongated fibroblast-like in appearance (Figures 2(b) and 2(c)), displaying larger spreading area compared to noncoated counterparts: although the cells could also attach to noncoated Non-PS and noncoated TCPS, they appeared in a smaller, more compact, and rounded morphology.

### 3.3. iMatrix-411 Stimulates MDPC-23 Cells Proliferation

**Figure 3:**
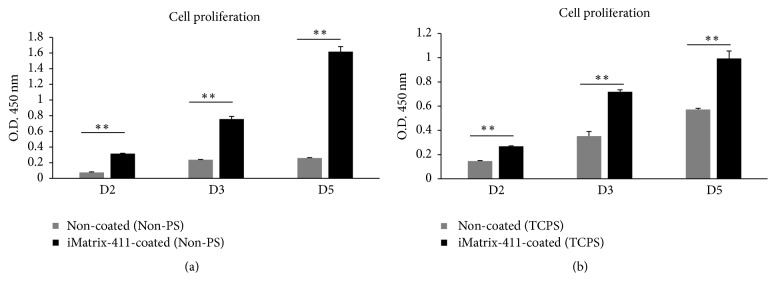
Cell proliferation on iMatrix-411-coated Non-PS (a) and TCPS (b). (a) Cell growth in iMatrix-411-coated Non-PS (black) and noncoated control (grey) at day two, day three, and day five (^*∗∗*^*p* < 0.01 by post hoc Tukey's HSD test). (b) Cell growth in iMatrix-411-coated TCPS (black) and noncoated control (grey) at day two, day three, and day five (^*∗∗*^*p* < 0.01 by post hoc Tukey's HSD test).

The effect of iMatrix-411 on proliferative activity of MDPC-23 cells was assessed by CCK-8 assay. On day two, day three, and day five, cell viability on iMatrix-411-modified Non-PS (Figure 3(a). D2: 0.32 ± 0.00 of iMatrix-411 versus 0.08 ± 0.01 of control; D3: 0.76 ± 0.04 of iMatrix-411 versus 0.24 ± 0.00 of control; D5: 1.62 ± 0.07 of iMatrix-411 versus 0.26 ± 0.01 of control) or TCPS (Figure 3(b). D2: 0.27 ± 0.00 of iMatrix-411 versus 0.15 ± 0.00 of control; D3: 0.72 ± 0.02 of iMatrix-411 versus 0.35 ± 0.04 of control; D5: 0.99 ± 0.06 of iMatrix-411 versus 0.57 ± 0.01 of control) was significantly elevated compared with noncoated controls. Furthermore, cells in noncoated Non-PS exhibited limited growth (Figure 3(a) grey), while those grown on noncoated TCPS (Figure 3(b) grey) adopted faster rate of growth.

### 3.4. iMatrix-411 Enhances ALP Activity

**Figure 4:**
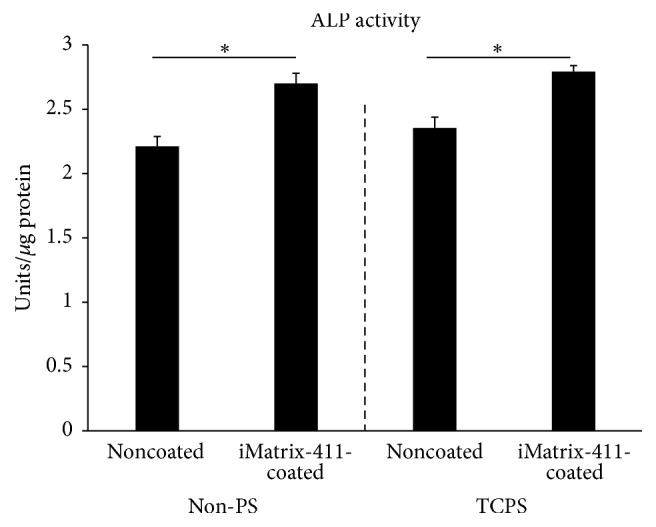
ALP activity of MDPC-23 cells cultured on iMatrix-411-coated Non-PS (left to dotted line) or TCPS (right to dotted line) was determined by a commercially available ALP kit assay (^*∗*^*p* < 0.05 by post hoc Tukey HSD test).

Cells grown on iMatrix-411 displayed significant higher ALP activity compared with those on noncoated controls (Figure 4). This enhancing effect applies to both Non-PS (Figure 4 left to dotted line: 2.70 ± 0.08 Units/*μ*g protein of iMatrix-411 versus 2.21 ± 0.08 Units/*μ*g protein of control) and TCPS (Figure 4 right to dotted line: 2.79 ± 0.05 Units/*μ*g protein of iMatrix-411 versus 2.35 ± 0.08 Units/*μ*g protein of control). However, ALP activity of cells seeded on noncoated TCPS did not differ with that on noncoated Non-PS (*p* > 0.05).

### 3.5. iMatrix-411 Triggers Upregulation of Osteo/Odontogenic Markers

**Figure 5:**
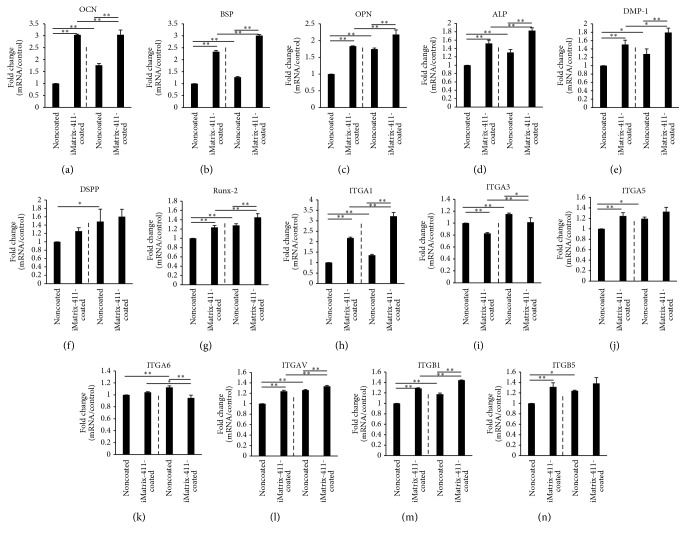
Gene expression level of osteo/odontogenic markers and integrins was quantitatively evaluated by real-time RT-PCR after total seven days of* in vitro* culture of MDPC-23 cells, with three days in mineralization medium. Significant enhanced gene expression levels in MDPC-23 cells growing on iMatrix-411-coated non-PS or TCPS could be detected for OCN (a) (3.03 ± 0.03-fold increase in iMatrix-411 modified Non-PS), BSP (b) (2.34 ± 0.05-fold increase in iMatrix-411 modified Non-PS), OPN (c) (1.83 ± 0.01-fold increase in iMatrix-411 modified Non-PS), ALP (d) (1.52 ± 0.08-fold increase in iMatrix-411 modified Non-PS), DMP-1 (e) (1.51 ± 0.10-fold increase in iMatrix-411 modified Non-PS), DSPP (f) (1.26 ± 0.08-fold increase in iMatrix-411 modified Non-PS), Runx-2 (g) (1.24 ± 0.04-fold increase in iMatrix-411 modified Non-PS); regarding integrins, except for ITGA1 (h) (2.17 ± 0.05-fold increase in iMatrix-411 modified Non-PS), the other six types of integrin were only mildly enhanced or downregulated by iMatrix-411. Dotted line in each panel divides the data into Non-PS (left) and TCPS groups (right) (^*∗*^*p* < 0.05, ^*∗∗*^*p* < 0.01, post hoc Tukey's HSD test).

The mRNA expression levels of seven types of osteo/odontogenic markers were evaluated. The control group (noncoated Non-PS) was set for the mRNA expression baselines (relative expression values at 100%). OCN displayed an increase of 3.03 times for MDPC-23 cells on iMatrix-411-modified Non-PS surface (Figure 5(a) left to dotted line). Moreover, seeding of cells in noncoated TCPS remarkably enhanced its expression to 1.77-fold compared with noncoated Non-PS (Figure 5(a) first and third bar). BSP, expressed by both osteoblast and odontoblast, demonstrated a 2.34-fold increase for the cells cultured on iMatrix-411-coated Non-PS compared with control (Figure 5(b) left to dotted line). Meanwhile, seeding of cells into TCPS could further elevate its expression by 1.27-fold (Figure 5(b) first and third bar). Both OPN (Figure 5(c): 1.83-fold increase versus control) and ALP (Figure 5(d): 1.52-fold versus control) expression levels for the cells on iMatrix-411-modified Non-PS were significantly higher than noncoated group as well. Similar to OCN, inoculation of cells into noncoated TCPS significantly enhanced the mRNA expression of both genes (OPN: 1.75-fold of increase in noncoated TCPS; ALP: 1.30-fold of increase in noncoated TCPS). For the remaining three genes, DMP-1 (Figure 5(e)), DSPP (Figure 5(f)) and Runx-2 (Figure 5(g)), there was only slight increment of expression on iMatrix-411 compared with control. Consistent with the above genes, gas plasma-treated noncoated TCPS promoted expression of these three genes compared with noncoated Non-PS.

### 3.6. iMatrix-411 Triggers Upregulation of Integrins

mRNA expression of seven integrins was quantified as well. Except for ITGA3 and ITGA6, expression of the other five integrins was found to be promoted by iMatrix-411. Specifically, ITGA1 was the one that was enhanced to the largest extent (2.17-fold increase, Figure 5(h)), also, cells cultured in noncoated TCPS promoted its expression to 1.35-fold (Figure 5(h) first and third bar). With regard to ITGA5, ITGAV, and ITGB1, they were promoted by iMatrix-411 to comparable levels in Non-PS (Figure 5(j) 1.25-fold, Figure 5(l) 1.24-fold, and Figure 5(m) 1.29-fold). Similarly, mRNA expression of the three integrins was elevated by seeding cells into noncoated TCPS compared with noncoated Non-PS. ITGB5, a reported fibronectin receptor, was enhanced by iMatrix-411 to 1.32-fold in Non-PS (Figure 5(n) left to dotted line). Seeding of cells to TCPS also augmented ITGB5 expression by 1.24 times, while no difference was detected in noncoated TCPS and iMatrix-411-coated TCPS (Figure 5(n) right to dotted line). In contrast, ITGA3 was downregulated by iMatrix-411 in Non-PS (0.83-fold) (Figure 5(i) left to dotted line); however, seeding cells to TCPS slightly upregulated its expression by 1.15-fold. Regarding ITGA6, there was no difference in expression between noncoated and iMatrix-411-coated Non-PS (Figure 5(k) left to dotted line); interestingly, in TCPS, it was found that the expression of ITGA6 was mildly suppressed by iMatrix-411 (Figure 5(k) right to dotted line).

### 3.7. iMatrix-411 Accelerates Mineralization on Nontissue Culture Treated-Polystyrene

**Figure 6:**
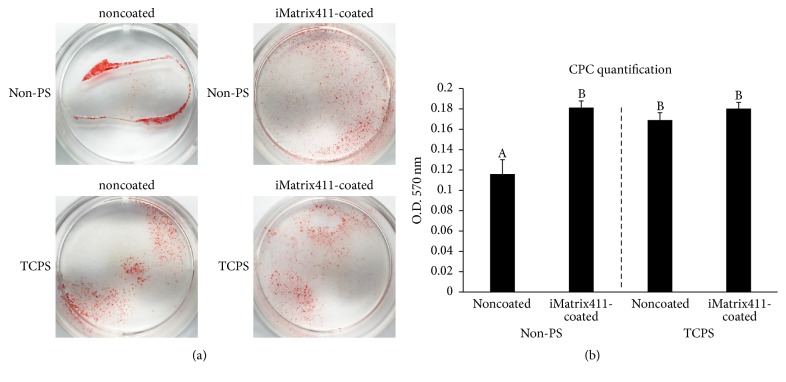
Alizarin red staining. (a) Mineralized nodules were stained using alizarin red s solution at day eight. (b) Quantification of staining intensity using CPC (A-B indicate significant differences between different characters, *p* < 0.01, post hoc Tukey's HSD test).

To evaluate the effects of iMatrix-411 in inducing a mature osteo/odontoblast phenotype, we stained the cells using alizarin red s to visualize the mineralized nodule formation on day eight.  Figure 6(a) shows that, in the Non-PS surface, iMatrix-411 significantly enhanced the mineralization of MDPC-23 cells as evidenced by the quantification data (Figure 6(b) left to dotted line). However, in TCPS surface, there was no difference with regard to the staining intensity between noncoated and iMatrix-411-coated groups (Figure 6(b) right to dotted line).

## 4. Discussion

Regulation of interactions between cells and extracellular matrix (ECM) lies at the center of such fundamental biological events as organogenesis. LN *α*4 chain was found to be expressed in tooth mesenchyme [19], while *β*1 and *γ*1 chains were expressed by the inner and outer enamel epithelium [20]. Given the proximity between LN-411 and tooth mesenchyme, it is therefore reasonable to conceive that there might be some interactions between this ECM protein and mesenchyme-derived odontoblasts. In the present study, to elucidate the influence of a novel fragment derived from LN-411 (iMatrix-411) in odontoblast-like cells, we analyzed* in vitro* the proliferation and differentiation of MDPC-23 cells to iMatrix-411 and searched for integrin receptors involved.

TCPS is widely used for mammalian adherent cell culture. Hydrophilic TCPS (water contact angle (WCA): 38° ± 9°) is processed from natural nonadhesive polystyrene (WCA: 84° ± 4°) [21] by gas plasma under vacuum. Adherent mammalian cells preferentially bind modestly hydrophilic surfaces exhibiting a WCA lower than 60° [22] and their contact areas were larger for those hydrophilic surfaces than for hydrophobic ones [23] Hence, in addition to examining the* in vitro* effects of iMatrix-411, we further investigated the cell parameters when they were cultured in both Non-PS and TCPS surfaces.

Cell proliferation was significantly increased in iMatrix-411-coated non-PS or TCPS. Growth of cells stagnated in noncoated Non-PS when seeded in low number (0.5 × 10^3^/well, 96-well plate), whereas adsorption of iMatrix-411 imparted this surface cell adhesiveness and promoted the growth of cells for three days tested. Simultaneously, the same tendency of cells grown on iMatrix-411-coated TCPS was observed. In contrast, despite the low seeding number, cells in noncoated TCPS did maintain a slower but steady growth compared with those on iMatrix-411-coated TCPS. Additionally, comparison of fold change regarding optical density revealed that a more drastic increase of cell proliferation activity exists between noncoated control and iMatrix-411-coated group on Non-PS substrates (Non-PS: D2: 4.16 times; D3: 3.17 times; D5: 6.22 times; TCPS: D2: 1.83 times; D3: 2.03 times; D5: 1.74 times). Indeed, although sequential events of cell adhesion including contact, attachment, spreading, and proliferation are similar among all surfaces, independent of surface chemistry, those events would be significantly delayed on hydrophobic surfaces [22]. Therefore, the hydrophilic TCPS was more adhesive than its Non-PS counterpart and leads to a less significant difference in proliferative activity of cells between noncoated and iMatrix-411-coated TCPS. Moreover, aside from iMatrix-411, there are a variety of proteins in serum that precipitated upon addition to the culture plastics. A study investigating the adsorbed fibronectin (FN) and vitronectin (VN) from serum revealed higher amount of both proteins was precipitated on TCPS but not Non-PS [24]. This might be another possible reason explaining faster growth of MDPC-23 cells in noncoated TCPS compared with noncoated Non-PS. Significant higher thickness of precipitated protein on TCPS (2.67 ± 0.11 nm) compared to Non-PS (2.11 ± 0.06 nm) [21] provides a possibility that it was due to this that leads to less differences of gene expression and cell proliferation data on TCPS.

Although Non-PS is nonadhesive, it still supports appreciable adherent cell growth, albeit to a lesser extent compared with TCPS. In particular, when the seeding number of cells is increased to a certain level, cells could grow in noncoated Non-PS wells to confluence. Cells were hence seeded at 1.25 × 10^4^/mL in 12-well plates and assayed for ALP activity. The results suggested iMatrix-411 leads to a higher ALP activity compared with control in both Non-PS and TCPS plates. As on day eight, the amount of total proteins was constant between groups (data not shown), the elevation detected in ALP activity is likely to be related to this enzyme and caused exclusively by the presence of iMatrix-411.

To further clarify whether upregulation of ALP activity in iMatrix-411 is related to the stimulation of osteo/odontoblastic differentiation, we quantified the expression of genes encoding markers of hard tissue forming phenotype. The molecular mechanisms of odontoblast differentiation were studied by examining the real-time RT-PCR. During teeth development, a number of genes are up- or downregulated to act synergistically or counteractively to fine tune the normal formation of dentin matrix. The most frequently used markers for odontoblastogenesis are as follows: DSPP, DMP-1, OCN, BSP, OPN, ALP, and Runx-2. As a parental protein for the predominant noncollagenous component (dentin phosphophoryn, DPP) in dentine matrix, DSPP is considered an unique hallmark for odontoblast differentiation. Although MDPC-23 cells were isolated from dental papillae and considered to be of odontoblast lineage, the real-time PCR Ct value for DSPP was around 30 (data not shown), meaning its* in vitro* expression level was quite low. Indeed, the* in vitro* 2D culture system differs significantly from the real condition; in situ differentiation of preodontoblast into functional, secretive mature odontoblasts requires robust epithelium and mesenchymal interaction. Here, iMatrix-411 showed limited capacity in inducing DSPP expression, revealing physical adsorption of a fragment derived from LN-411 into polystyrene was not adequate to support a phenotypic change of MDPC-23 cells into mature odontoblasts. Additional molecules such as BMP-2 or BMP-4 may help to synergistically act with iMatrix-411 in promoting DSPP expression. Furthermore, as noted above, a coculture system incorporating dental epithelial cells might also work; however, this hypothesis awaits future investigation. As the name indicates, DMP-1 was originally discovered in dentine matrix. Nevertheless, its function is not restricted to dentine; DMP-1 also initiates osteoblast differentiation and orchestrates mineralized matrix formation extracellularly at late stage of osteoblast maturation [25]. In this study, we found that iMatrix-411 promoted the mRNA expression of DMP-1 to a fold change that is similar to ALP. A comparison of all the osteo/odontogenic genes tested in the experiment indicated that OCN, a late stage osteoblast marker, was the one that was promoted by iMatrix-411 to the greatest extent. Besides its localization in the newly formed osteoid [25], the regenerative role of OCN in reversible pulpitis was highlighted by its presence in calcification sites and around blood vessels but not normal tissues [26]. Next to OCN, BSP and OPN were two elevated genes that increased by over 1.8-fold. Together with DMP-1 and DSPP, these four proteins (DMP-1, DSPP, BSP, and OPN) belong to small integrin-binding ligand N-linked glycoproteins (SIBLING) family. BSP is an early stage marker for osteoblast differentiation, while OPN peaks twice during the proliferation and differentiation of osteoblasts. Importantly, BSP and OPN were found to be predominantly expressed in reparative dentine, while DMP-1 and dentin sialoprotein (DSP) were expressed in primary dentine [27]. Taking the fold changes into consideration, it is thus suggested MDPC-23 cells cultured on iMatrix-411 assumed a phenotype more resembling osteoblasts rather than odontoblasts. Nevertheless, iMatrix-411 still holds a sound potential to be utilized in pulp capping treatment; as mentioned earlier, OCN, BSP, and OPN are all closely related to the reparative dentine formation. The alizarin red staining result further proved iMatrix-411 was effective in inducing the mineralization of matrix, which is an essential final step for the closure of injured exposed pulp.

Moreover, as LN supports many biological activities primarily by binding integrins at the surface of cells [28], we also investigated the mRNA expression levels of several integrins, namely, ITGA1, ITGA3, ITGA5, ITAG6, ITGAV, ITGB1, and ITGB5. Since LN-411 was discovered in the basement membrane of blood vessels [29], previous studies regarding the function of LN-411 were mainly focused on its roles in angiogenesis. The current study is the first one to show iMatrix-411, an integrin-binding fragment derived from LN-411, promotes the proliferation and differentiation in odontoblast-like cells. In this process, integrin *α*1 appears to play a significant role, as evidenced by the quantified gene expression data. Ozeki et al. reported integrin *α*1 was dramatically induced by a combination of retinoic acid and BMP-4 when culturing human skeletal muscle stem cells on gelatin and siRNA against integrin *α*1 completely blocked DSPP expression [30]. In addition, integrin *α*1 is a classical type I collagen receptor [31]. The upregulation of integrin *α*1 suggested that single integrin could bind with different ligands, acting as dual LN/collagen receptor. In fact, binding of integrins with extracellular matrix (ECM) proteins was found to be redundant and replaceable: knock-out of one integrin did not lead to severe life-threatening consequences [32]. In an earlier work, it was found that LN-411 used both integrin *β*1 and *α*v*β*3 to bind human dermal microvascular endothelial cells [33]. Integrin *α*3, a preferential receptor for LN-332 (or LN-5) LN-511 (LN-10) or LN-521 (LN-11) [34], was slightly downregulated by iMatrix-411. Due to different cell types and culture methods, differences in the integrin expression profile upon exposure to the same stimulant are not surprising. Integrins are transmembrane proteins. They primarily regulate cell attachment to ECM, a fundamental process that provides a dynamic physical linkage between the ECM and actin cytoskeleton. Engagement of integrins with ECM ligands triggers integrin clustering, which activates a number of intracellular signaling pathways to regulate cytoskeletal and ECM assembly and cell differentiation.

Except for direct comparison between noncoated wells and iMatrix-411-coated wells, we also compared the cell behavior in nontissue culture treated-polystyrene (Non-PS) and tissue culture treated-polystyrene (TCPS). Interestingly, we found that cultivation of cells in TCPS leads to a dramatic higher expression of OPN and OCN, denoting that they are more sensitive to the surface chemistry change than the other genes. Another paper reported the enhancement of mRNA encoding OPN and OCN following Ga-Al-As laser irradiation, which leads to production of reactive oxygen species (ROS) and hydroxyl radicals [35]. Our observation correlates well with their work because active oxygen groups were also generated by gas plasma treatment on TCPS. Aside from OPN and OCN, all the other genes were found to be enhanced in noncoated TCPS compared with noncoated Non-PS, albeit to a lesser extent. We attributed this generic observation to several causes: first, gas plasma treatment of TCPS increased its surface oxygen-containing groups (C-O and C=O) and the presence of those groups leads to reduced water contact angle and consequently increased hydrophilicity, thereby enhancing cell-substrates adhesion and proliferation [22]; that is, cell differentiation was accelerated than those on Non-PS because they reached confluence earlier; second, the hydrophilicity nature of TCPS leads to higher amount of adhesive matrix proteins precipitation from serum and synergistically facilitates more cell growth; third, cells secret various factors to sustain their growth and interaction with the external environment, with the increase of initial attached cell number; it is possible that they adopted much higher metabolic state and produce more proteins or factors and thereby further enhanced the differentiation. Therefore, because of the inherent hydrophilic nature of TCPS, it is shown in the data that the differences of some gene expression and mineralized nodule formation between noncoated TCPS and iMatrix-411-coated TCPS were not as much as that in Non-PS groups.

It needs to be pointed out that iMatrix-411 does not retain the conformation of its parental protein. The effects iMatrix-411 exhibited here may not reflect the comprehensive functions of LN-411. Site-directed mutagenesis and functional tests of modified LN-411* in vitro* and* in vivo* may be useful for further clarification. Nevertheless, our data provides novel insight into the* in vitro* effects of iMatrix-411 in odontoblast-like cell. It is suggested iMatrix-411 facilitates cell proliferation and enhances osteogenic differentiation. Further, the comparative study between Non-PS and TCPS indicates surface treatment by oxidation and promotes gene expression of key osteogenic markers.

## 5. Conclusions

By physically coating iMatrix-411 to non-PS or TCPS, the* in vitro* effects of MDPC-23 cells on the modified surfaces were evaluated. This study highlights an important issue that should be taken into consideration when selecting a proper culture substrate for* in vitro* experiments or tissue engineering applications. It was found that both the proliferation and differentiation of MDPC-23 cells were affected by using different types of polystyrene. This interesting phenomenon seems to have been ignored. Importantly, this work found that iMatrix-411 not only sustains the proliferation of MDPC-23 cells, but also contributes to the differentiation of the cells to odonto/osteogenic lineage through enhanced ALP activity and upregulated mRNA expression levels of key osteo/odontoblastic markers. These findings suggest that iMatrix-411 may provide a possible option for hard tissue regeneration. Moreover, because coating of iMatrix-411 on polystyrene surfaces resulted in enhanced cell proliferation and differentiation, this also suggests iMatrix-411 could possibly be used for surface modification of biomaterial scaffolds in dentin or bone tissue engineering.

## Figures and Tables

**Table 1:** Primer sequence, fragment size, and annealing temperature.

Gene name	Forward (5′ to 3′)	Backward (5′ to 3′)	Fragment size (bp)	*T*_*m*_
OCN	AGCTCAACCCCAATTGTGAC	AGCTGTGCCGTCCATACTTT	190	55
BSP	CTGCTTTAATCTTGCTCTG	CCATCTCCATTTTCTTCC	211	55
OPN	TTTCCCTGTTTCTGATGAACAGTAT	CTCTGCTTATACTCCTTGGACTGCT	228	55
ALP	GGAAGGAGGCAGGATTGACCAC	GGGCCTGGTAGTTGTTGTGAGC	338	55
DMP-1	CGTTCCTCTGGGGGCTGTCC	CCGGGATCATCGCTCTGCATC	577	60
DSPP	TCAATGGCGGGTGCTTTAGA	TGCTCACTGCACAACATGAAGA	111	62
Runx-2	CCACAGAGCTATTAAAGTGACAGTG	AACAAACTAGGTTTAGAGTCATCAAGC	87	55
ITGA1	TCAACGTTAGCCTCACCGTC	CAGGGATCGTCTCATTGGCA	396	59.9
ITGA3	GAAAGGCTGACCGACGACTA	TGCGTGGTACTTGGGCATAA	108	66
ITGA5	GAAGGGACGGAGTCAGTGTG	TGAATGGTGCTGCACTGGAT	127	66
ITGA6	CTGAGATCCACACTCAGCCG	GCATGGTATCGGGGAACACT	126	66
ITGAV	ATAAAGCGCGGATGGCAAAG	CTCACCCGAAGATAGGCGAC	213	64.9
ITGB1	ACAAGAGTGCCGTGACAACT	AGCTTGATTCCAAGGGTCCG	325	59.9
ITGB5	CACGGTCCATCATCTCTCGG	CATGGAGAGGGAGAGGTCCA	281	62.8
*β*-Actin	AACCCTAAGGCCAACAGTGAAAAG	TCATGAGGTAGTCTGTGAGGT	241	53

## Data Availability

All data generated or analyzed during this study are included in this article.
